# Detecting carotid stenosis from skin vibrations using Laser Doppler Vibrometry – An in vitro proof-of-concept

**DOI:** 10.1371/journal.pone.0218317

**Published:** 2019-06-20

**Authors:** Viviana Mancini, Daniela Tommasin, Yanlu Li, Jonathan Reeves, Roel Baets, Steve Greenwald, Patrick Segers, on behalf of the CARDIS consortium

**Affiliations:** 1 bioMMeda, Ghent University, Ghent, Belgium; 2 Department of Information Technology, Ghent University, Ghent, Belgium; 3 Blizard Institute, Queen Mary University of London, London, United Kingdom; 4 Clinical Physics, Barts Health NHS Trust, London, United Kingdom; Medical University Innsbruck, AUSTRIA

## Abstract

Early detection of asymptomatic carotid stenosis may help identifying individuals at risk of stroke. We explore a new method based on laser Doppler vibrometry (LDV) which could allow the non-contact detection of stenosis from neck skin vibrations due to stenosis-induced flow disturbances. Experimental fluid dynamical tests were performed with water on a severely stenosed patient-specific carotid bifurcation model. Measurements were taken under various physiological flow regimes both in a compliant and stiff-walled version of the model, at 1 to 4 diameters downstream from the stenosis. An inter-arterial pressure catheter was positioned as reference. Increasing flow led to corresponding increase in power spectral density (PSD) of pressure and LDV recordings in the 0–500 Hz range. The stiff model lead to higher PSD. PSD of the LDV signal was less dependent on the downstream measurement location than pressure. The strength of the association between PSD and flow level, model material and measuring location was highest in the 0–50 Hz range, however useful information was found up to 200 Hz. This proof-of-concept suggests that LDV has the potential to detect stenosis-induced disturbed flow. Further computational and clinical validation studies are ongoing to assess the sensitivity and specificity of the technique for clinical screening.

## Introduction

Stroke, a cardiovascular disease (CVD), is the second most common single cause of death in Europe [1]. There are many underlying causes of stroke, but one of the most prominent is carotid stenosis [2], as the atherosclerotic plaque causing the narrowing might rupture and the thrombi would obstruct the blood flow in downstream vessels. Unfortunately, few patients with carotid stenosis present symptoms in the early stages of the disease. Moderate and severe asymptomatic stenosis affect 12.5% and 3.1% of the over 70 population, respectively [3,4]. However, asymptomatic stenosis is not commonly diagnosed unless associated with other CVDs [5] such as coronary stenosis and hypertension, or risk factors such as diabetes [6,7]. Thus, the possibility of assessing and stratifying the risk of future stroke in asymptomatic patients with carotid stenosis is limited [8].

The presence of turbulence in stenosed carotid arteries has been established in numerous studies, see for example the results reported by Beach et al. [9] using ultrasonic duplex Doppler velocimetry *in-vivo*, by Giddens et al. [10] *in-vitro*, and by Lee et al. [11] and by Varghese et al. [12] by means of a computational fluid dynamics (CFD) approach. This turbulence may produce audible bruits induced by flow instabilities distal to the stenosis and carotid auscultation is currently used in clinical practice to detect carotid bruits. Although this is usually considered as an adequate screening test [8], it has a low-sensitivity [13], it is operator-dependent, and it is a subjective measurement that may be confounded by background noise [14]. Furthermore, carotid ultrasound, which is routinely used for stenosis diagnosis in hospitals, is too expensive and complex for use in first line screening.

Laser Doppler Vibrometry (LDV) has been shown to be particularly suitable for detecting physiological signals from skin level movements, for measurement of pulse wave velocity [15], breathing [16], or heart rate [17]. Within the context of an H2020 funded project (CARDIS), we have developed a compact multi-beam array prototype LDV system [18] based on optical chip technology [19] and suitable for use in a clinical setting for, among other things, measurement of arterial stiffness. Hypothesizing that post stenotic flow instabilities impart energy to the arterial wall, giving rise to mechanical waves which propagate through the soft tissues up to the skin, there is a potential use of LDV for the detection of stenosis in superficial arteries. The intended use for such device would be first line cardiovascular screening, identifying individuals at risk so that they can be referred to specialized centers for further follow-up using dedicated and established diagnostic techniques such as ultrasound or MRI.

The objective of the current work was therefore to provide a proof of principle concerning the ability of LDV to infer the presence of carotid stenosis. Specifically, we aimed to determine whether the presence of a stenosis leads to high frequency fluctuations in the recorded LDV signals and, if so, to assess the frequency range where such fluctuations would have to be sought. To do this, we performed hydraulic bench tests on a model of a patient-specific carotid bifurcation manufactured in two materials having different stiffness (referred to as compliant and stiff). The main purpose of the compliant model was to provide a physiologically plausible hemodynamic environment, allowing us to make a meaningful comparison between our findings and in-vivo data. On the other hand, the measurements on the stiff model were used to provide data for validation of a complementary computational rigid-wall approach, which will be presented elsewhere. The compliant and stiff models were subjected to pulsatile flow over a wide range of velocities. A catheter-tipped manometer was used as a reference for the LDV recordings. A frequency domain analysis of the signals was then performed to assess the impact of material stiffness and measurement location on the distribution of the energy content in the useful frequency range and multivariate linear regression analysis was used to determine the relative contribution of these factors to the relationship between volume flow rate and signal strength.

## Materials and methods

### Model reconstruction

**Fig 1:**
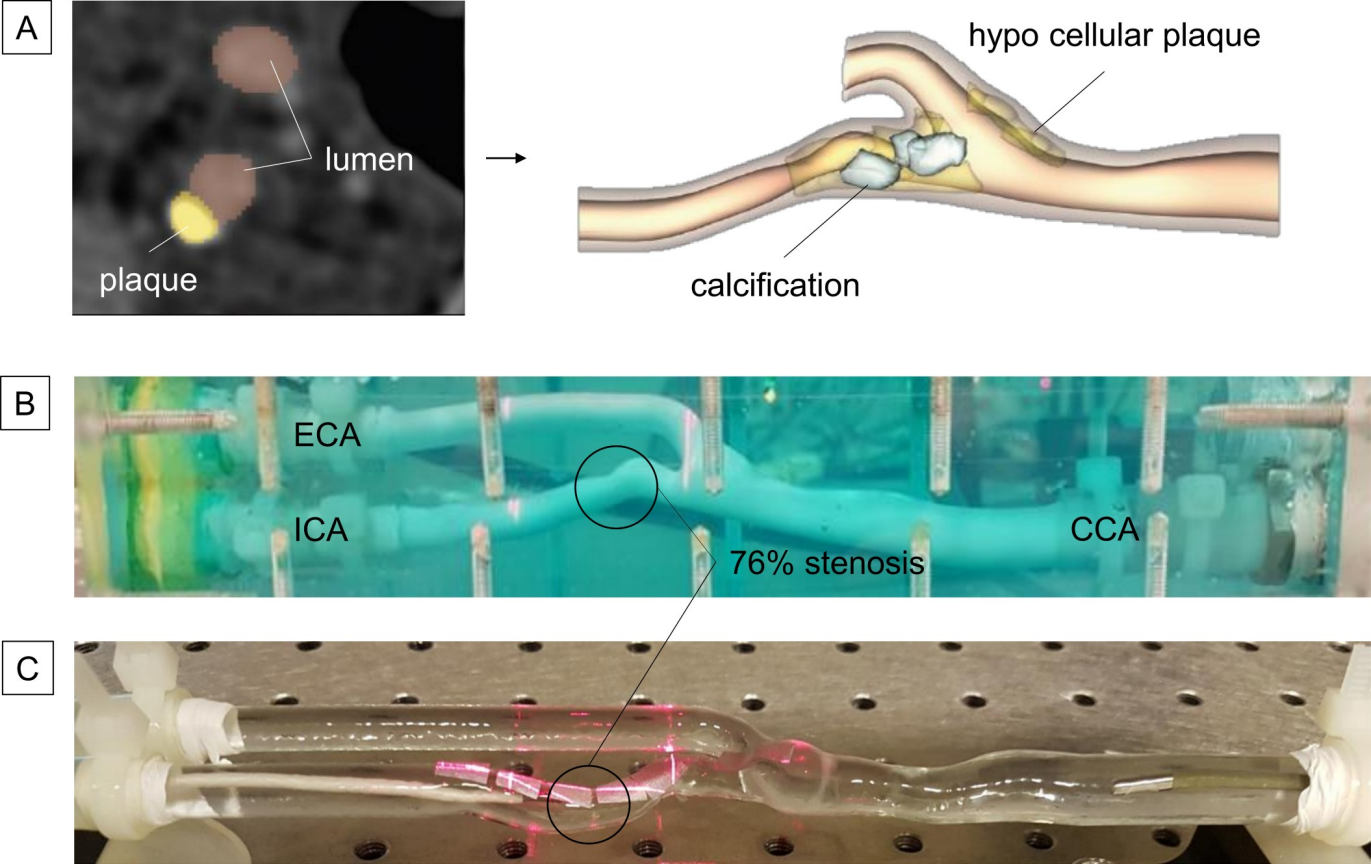
Patient-specific model. (A) CTA images of the carotid bifurcation from a 75 year old male were processed by Iannaccone et al.[20] in order to obtain a patient-specific model of the plaque-free stenosed vessel. The compliant (B) and stiff (C) models used for the hydraulic tests replicate the geometry of the patient-specific common carotid artery (CCA), external carotid artery (ECA), and internal carotid artery (ICA) where the 76% area stenosis (circled) is located.

Images of the common carotid bifurcation with severe internal carotid artery (ICA) stenosis of a 75 year old man were obtained by computed tomographic angiography (CTA), the patient having provided informed consent for the processing and further use of the data. The images were segmented by means of 3D Slicer [20], to obtain an accurate model of the vasculature (Fig 1A). The degree of stenosis of the carotid model was 76% as calculated by the NASCET method [21], where A_stenosis_ = 5.57 mm^2^ and the ICA area downstream of the stenosis was A_downstream_ = 23.14 mm^2^.

A compliant model of the carotid bifurcation was created in silicone rubber, as follows. A brittle inner mold of the bifurcation was 3D printed in VisiJet-pXL and sprayed with a finishing ColorBond (3D System, SUA) bonding layer (TRIAXIS, Belgium) in order to smooth the inner surface of the model. In our laboratory, liquid silicone (Xiameter rtv-481, De Gouden Pluim, Belgium) was poured around the mold and allowed to dry for 24 hours. The pouring was repeated until the model was thick enough to be handled; three layers being found sufficient. The inner mold was crushed and the bonding layer allowed an easier extraction of the crumbled pieces. Tensile tests were performed on samples the thickness of which was comparable with that of the carotid model (1.5mm), and they revealed a Young modulus (E) of 300±2kPa. The silicone model was then mounted in an open-topped Perspex box and surrounded by an aqueous hydrogel (Aquasonic 100, Parker Labs, USA) to mimic the neck’s soft tissues (see Fig 1B). A skin-like layer (polyurethane foil, Platilon, Epurex Films, Germany) was then applied over the gel surface. To ensure adequate reflection of the laser light [19], small patches of retro-reflective tape (3M Scotchlite High Gain Reflective Sheeting 7610, USA) were attached to the skin layer.

A second model with the same arterial lumen geometry was then 3D printed in a stiff transparent and water-resistant material (TuskXC2700T, Materialise, Belgium; E = 2700MPa (manufacturer data)), Fig 1C. The stiff model was used to provide data for a computational fluid dynamical model, described in [22], in which the presence of the gel was not taken into account. The small patches of retro-reflective tape were attached directly to the outer surface, omitting the use of the gel.

### Experimental set-up

**Fig 2:**
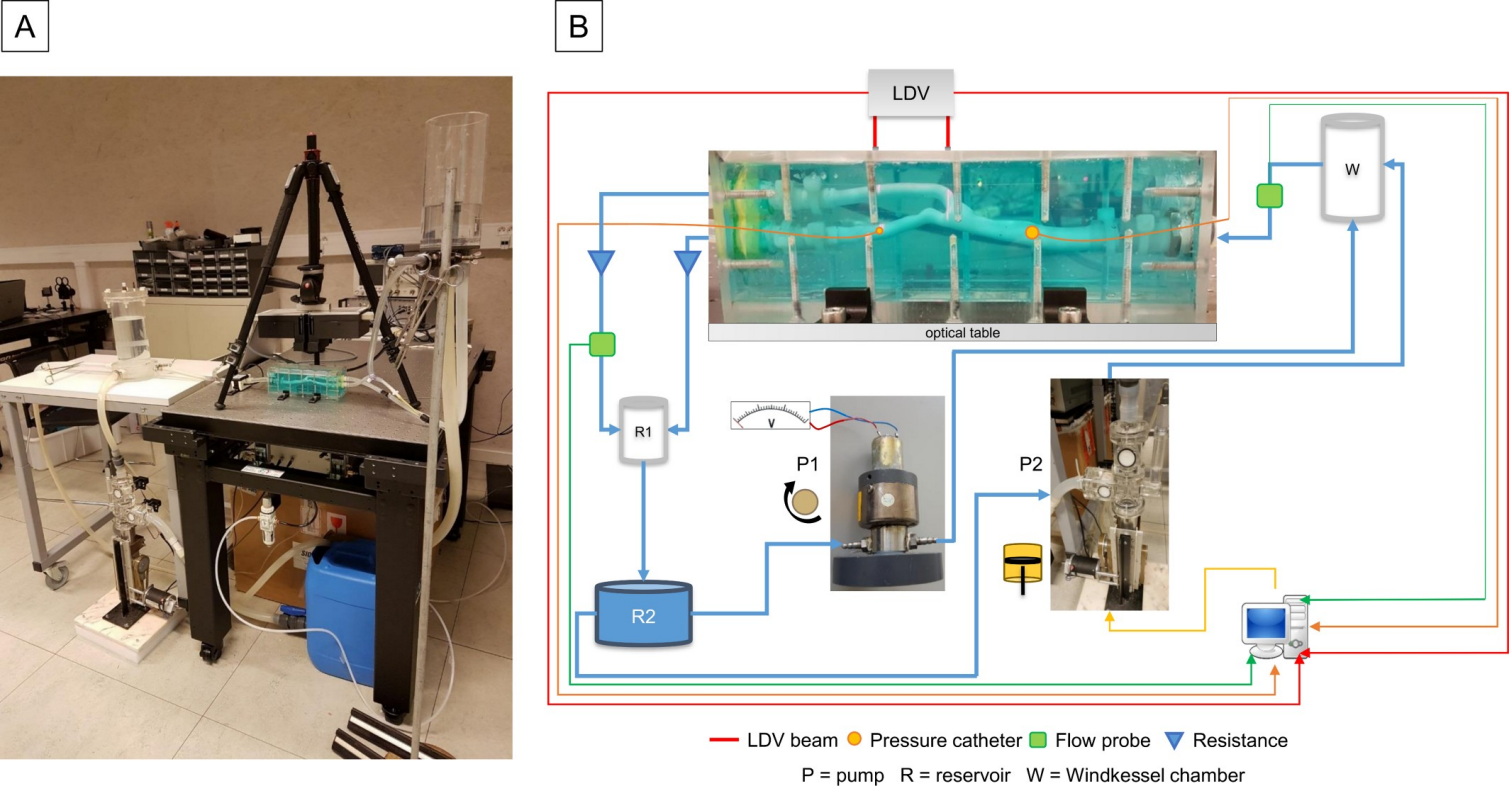
The assembled loop. (A) The model was secured to an optical table in order to minimize the influence of external movements. (B) In this layout of the test rig, the flow is depicted as a blue line where the arrow represents the direction of the flow. The thinner red, orange and green lines represent the signal acquisition wires of the LDV, pressure probes and flow probes, respectively. This set-up was used for both the compliant and stiff models.

A prototype multi-beam LDV [23] was used for all recordings. The device was designed with two parallel beam rows spaced 2.5, 3.5 or 5cm, configurable during assembly. Each row consisted of six beam points, spaced 5mm from each other. The laser wavelength was measured to be 1550±2nm. The temporal resolution was 10μs at a sample rate of 100kHz. The maximum measurable velocity was higher than 50m/s and the displacement resolution was less than 10nm. A calibration measurement between DC and 1kHz showed that the frequency response of the LDV was flat in this frequency range. In the recordings described here, only two beams were used. The LDV was mounted on a tripod and the models were mounted on a stabilized optical table and connected to a perfusion loop as shown in Fig 2A.

A schematic representation of the hydraulic setup is shown in Fig 2B. The loop was composed of a continuous flow pump (P1) and a pulsatile counterpart (P2), a Windkessel chamber (W), and two reservoirs (R1, R2). P1, a rotary pump (Gear Pump Head V108.05R Ryton Gears, Verder NV, Belgium), provided continuous flow, whose magnitude was controlled by varying the supply voltage, while P2, a custom-built programmable piston pump (HT Denyer, Consulting Engineer, UK), provided pulsatile flow. The speed and stroke of the piston and the opening and closing time of “mitral” and “aortic” valves were set by means of in-house software (HPUMP Controller v0.4b). The pumps could be controlled independently and could therefore run simultaneously or individually. Both pumps supplied flow to the Windkessel chamber which, in the case of pulsatile flow, would then deliver a dampened flow waveform to the common carotid artery (CCA) of the mounted model. A cannulating ultrasonic flow probe (ME 5PXN, Transonic Systems Inc., USA) was installed between W and the model. A catheter tip manometer (D.CTC/6F-1, Gaeltec Devices Ltd, UK) was passed into the CCA with the tip located 6.5 cm upstream of the center of the stenosis. A second, smaller, manometer (12-CTC/4F-1, Gaeltec Devices Ltd, UK) was passed into the ICA and positioned at one of four locations downstream from the center of the stenosis. The first location was one CCA diameter (0.8cm) downstream and each of the following locations was an additional CCA diameter downstream of its predecessor. The four locations are, from here on, referred to as 1D, 2D, 3D and 4D. The downstream beam of the LDV was moved horizontally together with the small manometer to record, respectively, skin displacement and intra-arterial pressure at the same horizontal location. The upstream LDV beam recorded vibrations 3.5cm upstream of the small pressure probe. The flow rate through the ICA and external carotid artery (ECA) branches was controlled by increasing or decreasing the resistances in the lines connecting each of the branches to the downstream reservoir R1, the purpose of which was to provide a constant back-pressure to the model. A second ultrasound-based flow probe (ME 4PXN, Transonic Systems Inc., USA) was installed downstream of the model, in the line connecting the ECA to R1. The water was then collected in the further downstream reservoir R2, which then supplied fluid for both pumps P1 and P2. The pipes connecting the elements of the loop were of compliant material (silicone rubber SL601).

### Flow conditions

P1 and P2 could be combined and their output adjusted to match physiological conditions [24,25]. We aimed for a physiological mean flow rate of 337 ml/min and four other flow rates corresponding to the mean value plus and minus one and two standard deviations (SD), as derived from the data in [24], giving values of 433, 241 (±1SD) and 529 and 145 (±2SD) ml/min.

The flow split was set to mimic physiological conditions as well [26], where we aimed for default values of 55% to the ECA and 45% to the ICA. We then varied the ICA flow split within the range 0.45±0.13. The flow split values were set when Q_CCA_ = 337ml/min, but they did not change greatly when the Q_CCA_ changed. This standard setting resulted in a pulse pressure (PP) of about 19mmHg for a heart rate of 60beats/minute. Additional experiments were then performed to enforce a PP_CCA_ of 40mmHg, which could only be achieved with supra-physiological flow levels (around 650ml/min). As the stiff model was able to sustain higher pressures, we tested flow rates up to 1465ml/min for that model. In all, the test conditions span a wide range of flow levels, encompassing the physiological range, both below and above levels expected to trigger flow instabilities. Additionally, the pressure catheters were, from time to time, removed from the main loop in order to test the impact of their presence on the flow.

**Fig 3:**
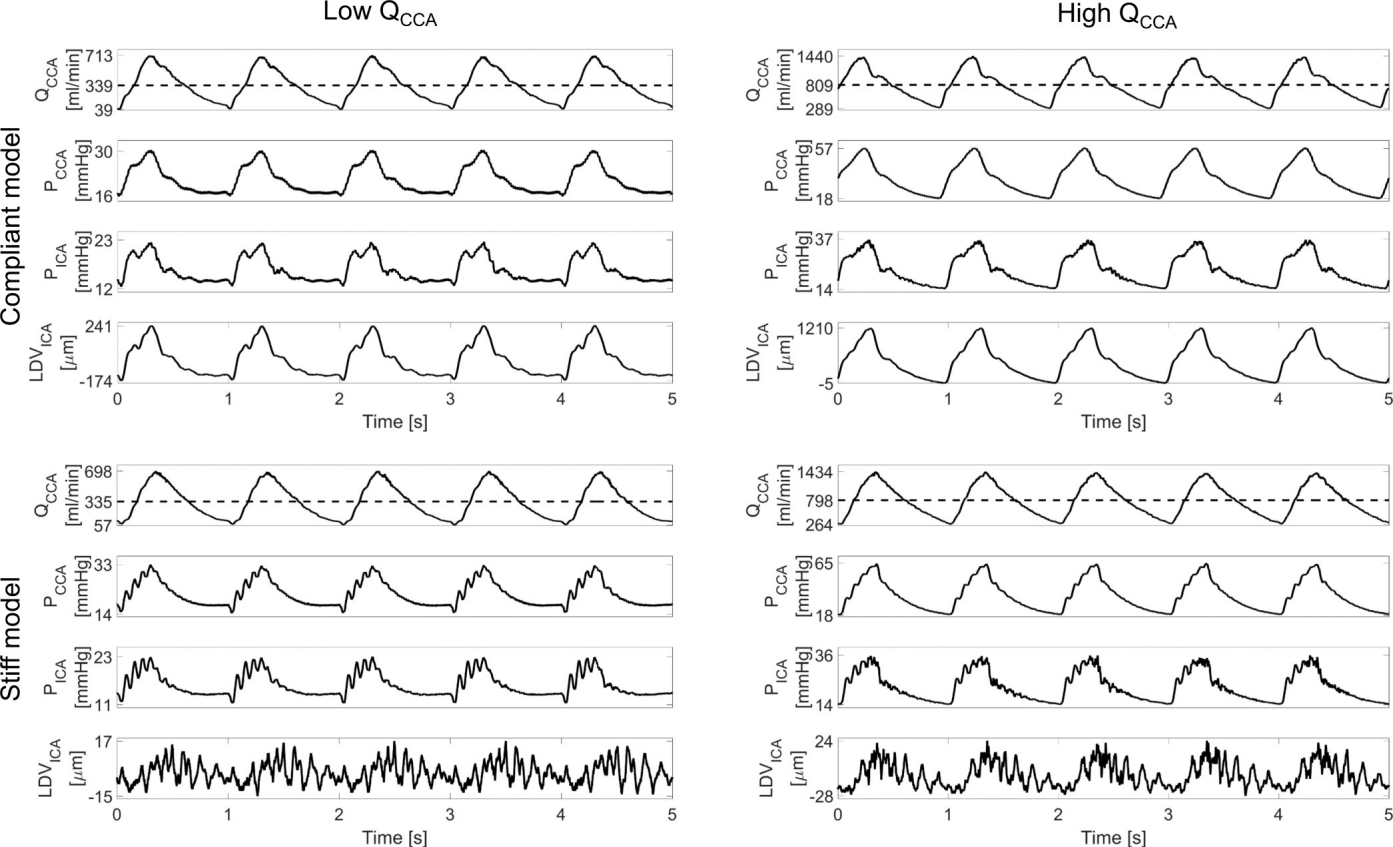
**Time traces** for the compliant (upper panels) and stiff (bottom panels) models obtained at 1D with low (left panels) and high (right panels) **Q**_**CCA**_. The horizontal dashed line shows the mean **Q**_**CCA**_.

The signals, recorded by the six probes (two LDV beams, two pressure probes and two flow probes), were acquired by means of Powerlab-16/35 (AD Instruments, Oxford, UK) at a sampling frequency of 20kHz. Once the system provided stable fluid dynamic conditions, data were recorded for at least 10 seconds and saved via dedicated software (LabChart Pro-v8). Some representative signals are shown in Fig 3. The left panel of Fig 3 depicts the waveforms of Q_CCA_, pressure at the ICA (P_ICA_) and LDV at the same horizontal location as the tip of the manometer (LDV_ICA_) for physiological mean flow rate and flow split settings, (i.e. QICA/QCCA≅45%), for both the compliant (upper left) and stiff (lower left) models. While the shape of the flow waveforms differs only in the presence or absence of the dicrotic notch, the shapes of the pressure and LDV signals in the two models differ drastically. Note the small amplitude of the LDV signal when compared to that measured in the compliant model. The same considerations can be applied to the waveforms obtained by setting Q_CCA_>700ml/min, as shown in the upper right and bottom right panel of Fig 3 for the compliant and stiff models, respectively. When increasing the flow rate, however, the waveforms shapes differ as well. Moreover, the high-frequency content of the pressure signal P_ICA_ is more evident.

### Signal processing

#### Power spectral density

**Fig 4:**
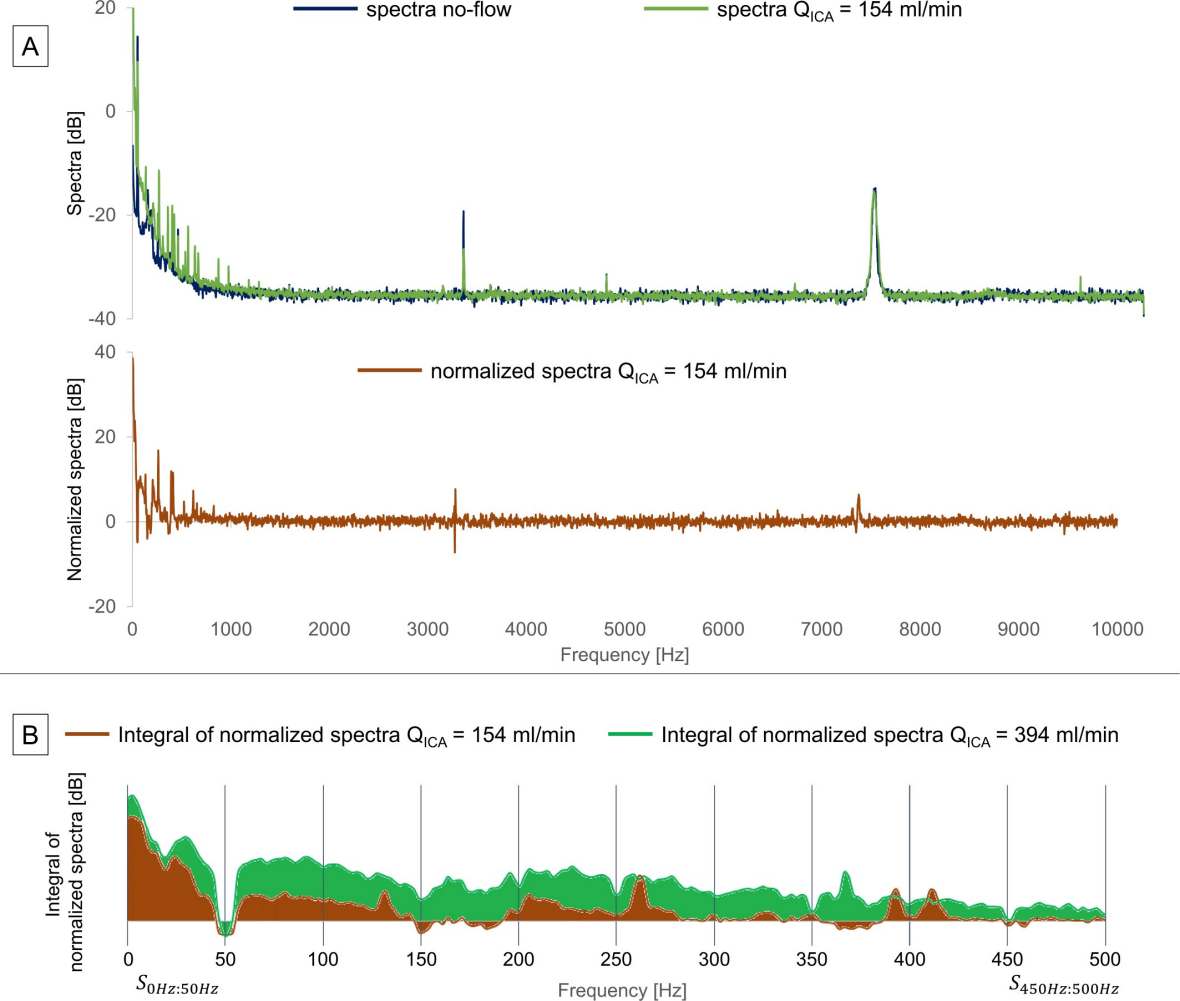
Workflow of the signal processing. (A) The spectrum of the no-flow pressure signals was subtracted from the spectrum of each flow recording to obtain the normalized spectrum, (B) its integral, based on the trapezoidal method and (C) the sum of the integrals in 50 Hz-wide frequency ranges, (D) the same post processing was applied to all available recordings.

Frequency spectra of the recorded data, obtained by the fast Fourier transform (FFT), were computed using LabChart Pro-v8. 8K points were used for the FFT with 50% overlap and Hann windowing [27]. The spectrograms were calculated by means of the same software with 1K points, Hann windowing with 93% overlap. The Power Spectral Density (PSD) represents the amount of energy present in the analyzed signals at each point in the frequency domain [28]. We calculated the PSDs with reference to an attenuation value of 0.001 units for all measurements, and it is hence expressed in decibels (dB). The spectra were then imported to Excel (Microsoft, 2016) for further processing. Fig 4 shows the processing steps for the P_ICA_ signal shown in the upper left corner of Fig 3 (mean Q_ICA_ = 154ml/min). The spectrum of the P_ICA_ (Fig 4A, upper panel) is shown against that of the no-flow condition in the available frequency range (hence 10kHz, based on a sampling frequency of 20kHz). The intense ~3500Hz and ~7500Hz peaks were present in the spectra of both the no-flow and the Q_ICA_ = 154ml/min signals. The subtraction of no-flow spectra made it possible to reduce the noise component of the Q_ICA_ = 154ml/min pressure spectrum, as shown in the bottom of panel A. The absolute value of the normalized Q_ICA_ = 154 ml/min spectra was shifted by the value of the no-flow one, from an average of ~ -30dB to ~0dB. All the signals were processed with this method, hence the shifting was applied to all the spectra. To quantify the energy within defined frequency bands, we calculated the integral of the normalized spectra in the 0-500Hz range by means of the trapezoidal method. The I_n_ integrals were grouped in Δf = 50 Hz wide frequency intervals and their sum was calculated as $S_{n:n+\Delta f}=\sum_{f=n}^{n+\Delta f}I_{f}$. The integral calculation of the normalized Q_ICA_ = 154ml/min and Q_ICA_ = 394ml/min (matching with signal P_ICA_ of Fig 3 upper right) spectra is shown in Fig 4B. The same workflow was applied to all available recordings, for both pressure and LDV in the ICA.

#### Statistical analysis

To assess the ability of LDV to recognize different flow levels with different materials, pump settings and probe locations, and hence to assess the frequency band where the spectra are most sensitive to the change in flow rate, further statistical analysis was necessary. We therefore constructed a univariate linear model in IBM SPSS Statistics 25, with the integrals of the 50Hz wide normalized spectra intervals, with downstream P_ICA_ and LDV_ICA_ signals, as dependent variables. Q_ICA_ was used as a covariate and the frequency range as a fixed factor (0 ≡ 0-50Hz, 1 ≡ 50-100Hz, …, 9 ≡ 450-500Hz). Additionally, location (1D ≡ 1, 2D ≡ 2, 3D ≡ 3 or 4D ≡ 4), material (compliant ≡ 1 or stiff ≡ 0) and the impact of the presence of the intra-arterial pressure probe on the flow field (present ≡ 1, not present ≡ 0) were used as fixed factors. As we wished to study the extent to which an increase in flow increases the power within a certain frequency band, we also added the interaction term, *Freq*^.^*Q*_*ICA*_, to the model. The resulting univariate model to evaluate the area under the curve (*AUC*) is expressed in Eq (1).

$$AUC=constant+A∙Freq+B∙Location+C∙Material+D∙Probes+E∙Q_{ICA}+F∙Freq∙Q_{ICA}$$ (1)

The estimated F coefficient was used as measure of sensitivity, since it reflects the slope of the regression equation for predicting the dependent variable from the independent one. P-values *p < 0*.*01* were considered significant.

Additionally, the Estimated Marginal Means (EMMs) were computed for both P_ICA_ and LDV_ICA_ in the 0-500Hz range. The EMMs are the variable’s means adjusted for the other variables of the group, they hence take into account statistically, the influence of the other variables on the calculation of the adjusted mean. The Bonferroni adjustment, used here [29], is one of several methods used to counteract the problem of multiple comparisons.

## Results

In total, 564 data sets were recorded, with about 50% of the experiments performed under physiological flow conditions. An overview of how many experiments have been conducted under which condition is provided in Table 1. We provide the raw data in a public repository [30]. The resulting Reynolds number (Re) of the ICA, based on the flow through it (Q_ICA_), its cross sectional area A_ICA_ = 8.74 mm^2^ at the bifurcation and on the kinematic viscosity of water of 1 ^.^ 10^−6^ m^2^/s, ranged from 198 to 5774.

**Table 1:** Overview of test conditions with the number of recordings (N), mean CCA pulse pressure (PP_CCA_) and flow rate (Q_ICA_) in the internal carotid artery.

Model	N	PP_CCA_ (mmHg)	Q_ICA_ (ml/min)
**Physiological flow rates**			
**Compliant**	55	16.07 ± 6.62	149.74 ± 77.97
**Stiff**	197	20.02 ± 8.07	121.88 ± 84.61
**Physiological pulse pressures**			
**Compliant**	40	39.92 ± 3.35	348.3 ± 59.91
**Stiff**	146	42.78 ± 4.23	302.41 ± 69.19
**Extreme settings for stiff model**			
**Stiff**	125	98.39 ± 25.43	585.81 ± 138.17

### Spectrograms

**Fig 5:**
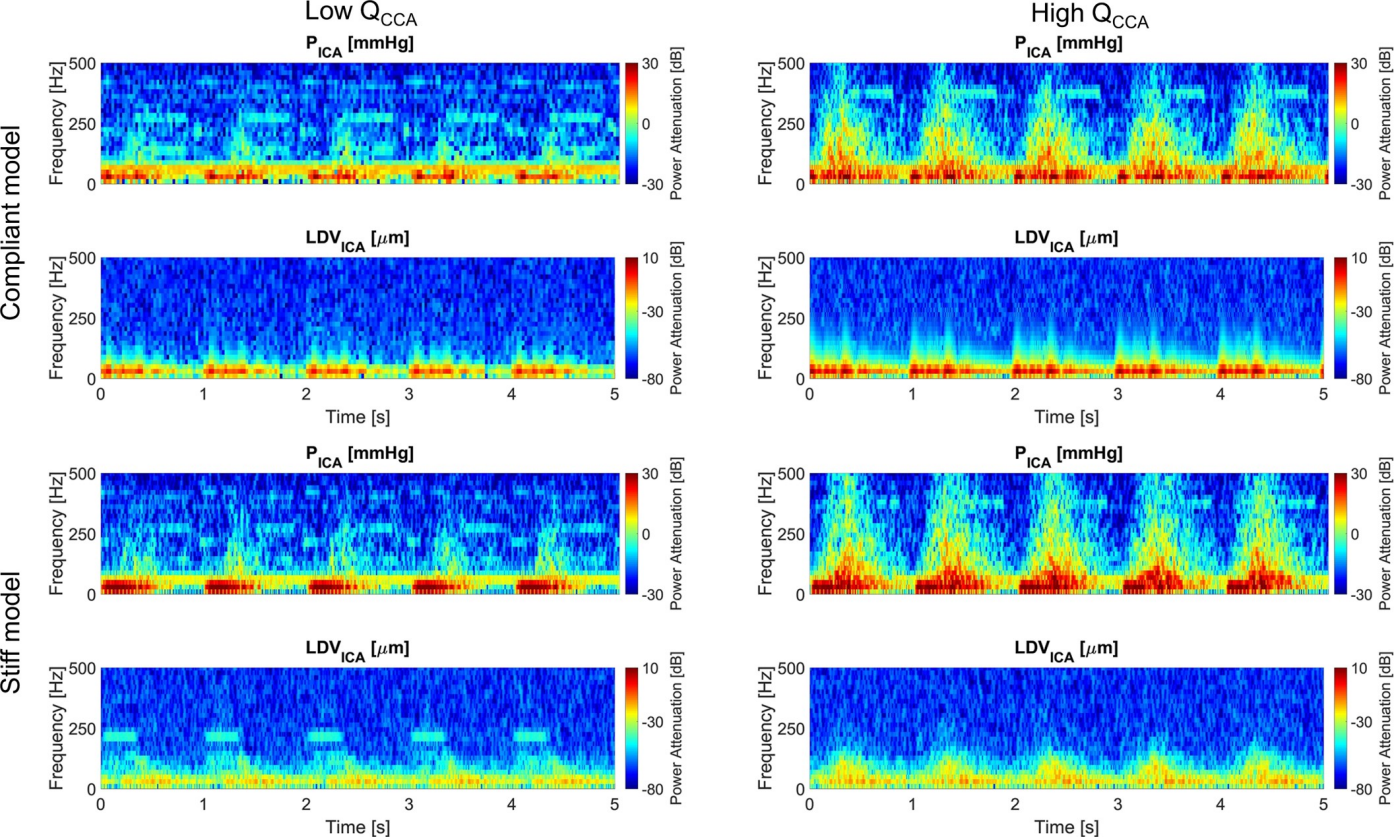
**Spectrograms** of the pressure and LDV at the ICA for the compliant (upper panels) and stiff (bottom panels) models obtained at 1D with low (left panels) and high (right panels) **Q**_**CCA**_.

The spectrograms of the P_ICA_ and LDV_ICA_ signals previously shown in Fig 3, are presented in Fig 5 in the 0-500Hz range. The cardiac pulsation is evident in both the pressure and LDV signals. In the left panels (low flow rate), the highest intensity is around 50Hz extending up to ~250Hz (more prominent in the pressure signal) when the pressure and LDV reached their peak in the time domain and during the following deceleration phase. For the higher flow rates (right panels) the high-intensity and medium-intensity regions (red and yellow shading) move towards higher frequency values. However, little energy content was present above 500Hz. The maximum intensity of the LDV signal spectra are, as expected, lower for the stiff model than for its more compliant counterpart (bottom panels).

### Normalized spectra

**Fig 6:**
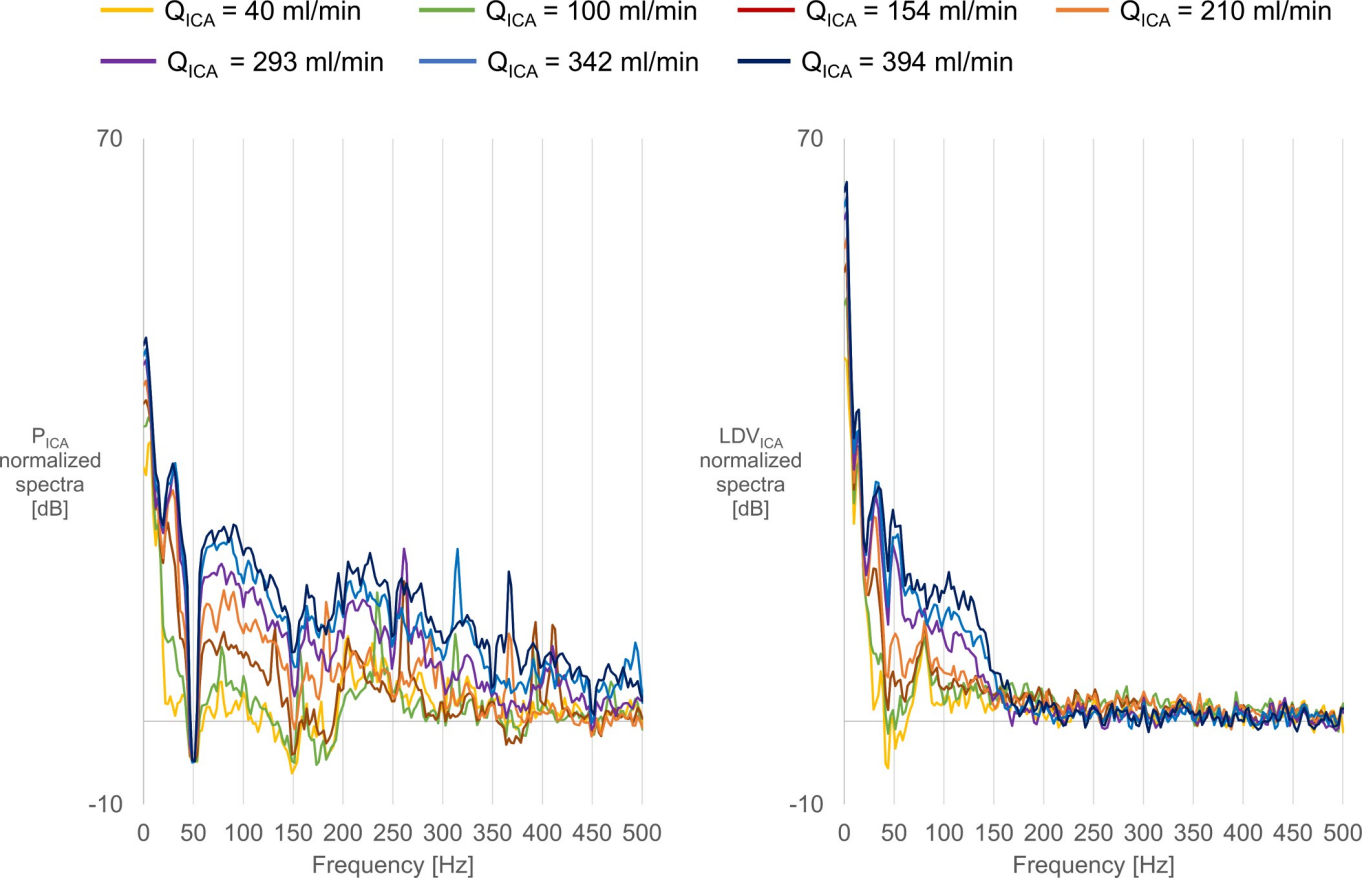
**Normalized spectra** of pressure (left panel) and LDV (right panel) signals, for multiple **Q**_**ICA**_ in the 0–500 Hz range.

The normalized spectra of downstream pressure and downstream LDV signals, at location 1D for the compliant model, are depicted in Fig 6 in the left and right panels, respectively.

The spectra of the pressure signal showed a marked tendency to increasing energy content when moving towards higher flows. One aberrant finding is the drop-out near 50Hz of the pressure signal. This may be explained by the presence of appreciable electrical noise in the pressure recordings, especially under no-flow conditions, thus reducing the amplitude when normalizing the pressure spectra by subtracting the zero flow spectrum.

The spectra of the LDV signal showed, as well, a marked tendency to increasing energy content while moving towards higher flows. A notable finding was that the LDV was not affected by the 50Hz electrical noise. The spectra followed a similar trend for all flows, except for a 78Hz peak which was present for Q_ICA_<250 ml/min while it broadened in the 100-150Hz range for higher flow levels. The amplitude of the LDV spectra dropped for all flows around 150Hz, and no meaningful signals could be detected above this frequency. The LDV signals showed the same pattern of increasing energy with increasing Q_ICA_ as those of the intra-arterial pressure.

### Sensitivity analysis

**Fig 7:**
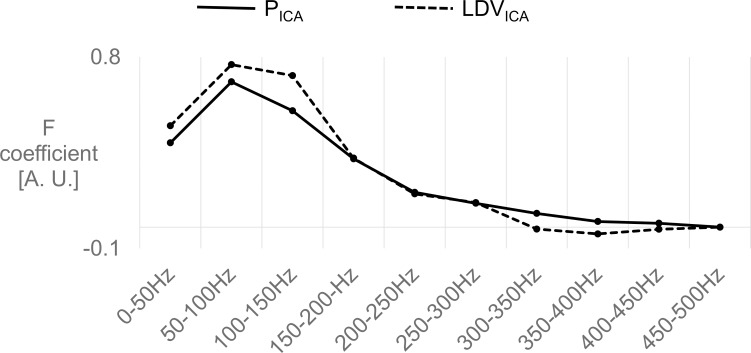
The F coefficient of *Freq*^.^*Q*_*ICA*_, resulting from the regression analysis of all available signals, showed that the sensitivity of LDV and pressure have a similar trend and that their sensitivity is the highest in the 50–150 Hz range.

The statistical analysis performed for downstream pressure (P_ICA_) and downstream LDV (LDV_ICA_) signals yielded the F coefficient of the *Freq*^.^*Q*_*ICA*_ term, which revealed a similar trend for both signals with a peak in the 50-150Hz range (Fig 7), where the sensitivity of the LDV was higher than that of the pressure catheter.

To compare LDV and pressure measurements, the EMMs (as a measure of the effect of the other variables on the calculation of the adjusted means) were evaluated when the pressure probe was inserted in the model, meaning evaluating Eq (1) for condition *probes = 1*.

#### Compliant vs. stiff model

**Fig 8:**
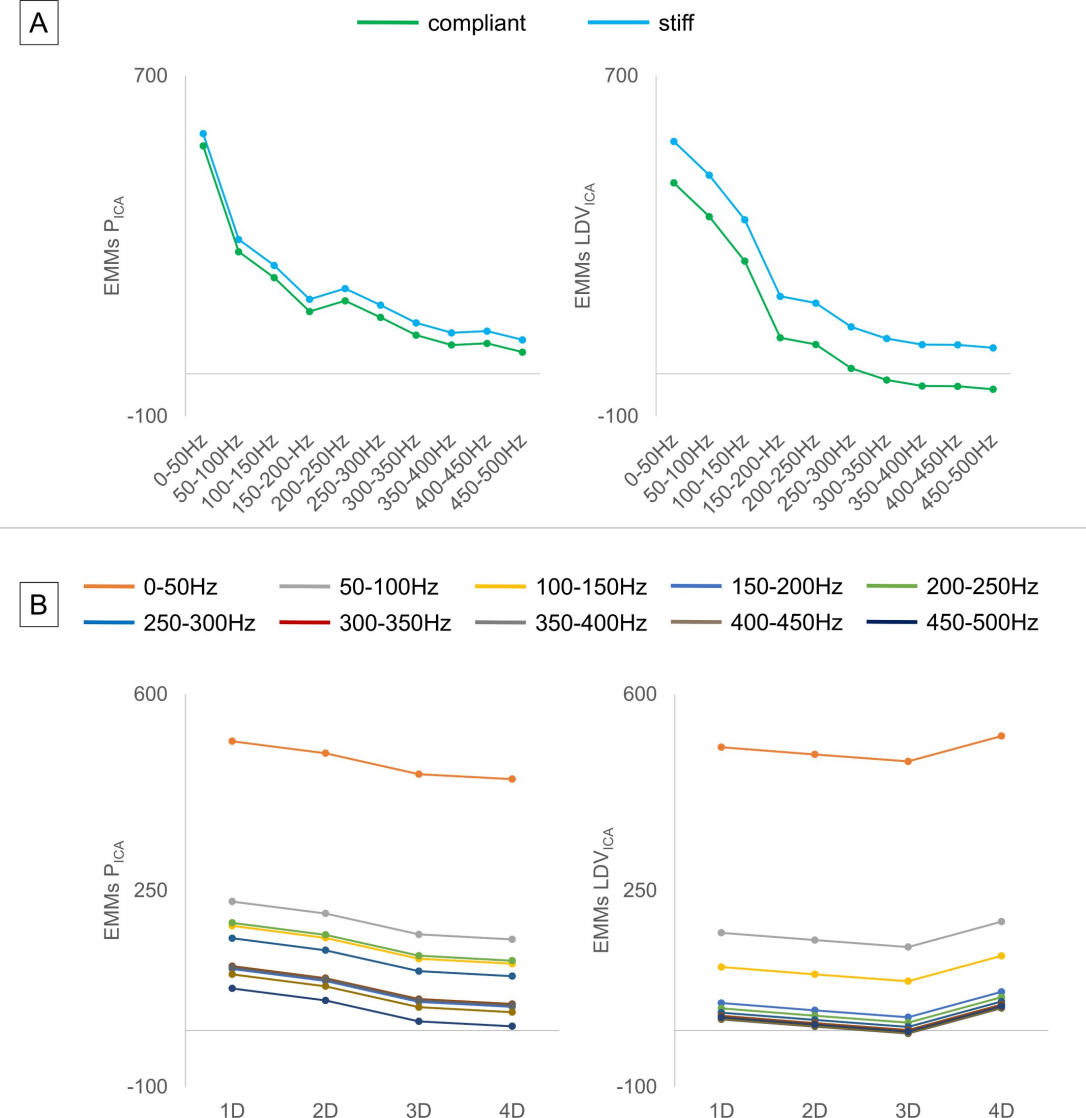
Estimated Marginal Means (EMMs). (A) The stiff model lead to higher EMMs for both the integrals of normalized spectra of pressure and LDV in the ICA (P_**ICA**_ and LDV_**ICA**_, respectively). (B) The EMMs decreased while moving further downstream from the stenosis for the pressure, and this trend was not as marked for the LDV where the 4D signal was above the 1D level.

The EMMs were evaluated regardless of the location in order to assess the impact of the model’s material. The total number of samples for each frequency range was N = 357 and N = 49, for the stiff and compliant model, respectively. The Q_ICA_ was 288.31±201.35 ml/min. The model material had an effect on the overall dependency of the relation between PSD and frequency and the flow level, but this effect was similar for the pressure and the LDV measurements (*p < 1*.*0E-06*, *p < 1*.*0E-06*), as shown in Fig 8, left and right upper panels, respectively. The EMMs of the stiff model were higher than those of the compliant model for both signals, and the difference between them was more marked in the LDV.

#### Measurement location

To evaluate the impact of the measurement location on the LDV and pressure signals, we conducted an analysis for the compliant model only because of its greater clinical relevance. The resulting Q_ICA_ was 230.34 ± 119.89 ml/min, with N = 14, 13, 10 and 12 for each 50 Hz-wide frequency band, for measurement locations 1D, 2D, 3D and 4D, respectively. The EMMs of pressure decreased as the distance from the center of the stenosis increased (*p < 1*.*0E-06*), for all frequency bands (Fig 8 –left bottom). The strongest signal was found at 1D downstream from the stenosis. The gap between the 0–50 Hz range and the higher frequency bands is noteworthy. The maximum was in the 0–50 Hz range for the LDV as well (Fig 8, right bottom). For the LDV, however, the EMM value at 4D was found to be higher than at 1D. Therefore, for the LDV data, measurement location was not significant (*p > 0*.*01*).

However, it is relevant to note that, despite their small size (6F and 4F), the presence of pressure probes in the models was found to be a statistically significant factor (*p < 0*.*01*). The impact of the measurement location in the compliant model for the LDV was found to be significant (*p < 0*.*01*) but only when the manometers were not present, i.e. *probes =* 0.

## Discussion

In this hydraulic bench model study of a carotid artery, we have shown that post-stenotic flow instabilities induce vibrations that can be detected from surface measurements by means of laser Doppler vibrometry. Frequency domain analysis revealed that the relevant frequency band is 0–200 Hz, as little useful information was found at frequencies above 200 Hz. The sensitivity of the LDV measurement to changes in trans-stenotic flow level was found to be comparable to what can be achieved using an intra-arterial manometer. We consider the results of our study a proof-of-concept of the technique for carotid stenosis diagnosis by detecting neck skin vibrations.

The main purpose of these tests was to establish whether the LDV was able to distinguish different trans-stenotic flow regimes (and presumed different levels of flow instabilities) and to determine the relevant frequency range to investigate. To quantitatively address these questions, a statistical analysis of the energy content of the LDV and reference pressure signals was conducted by applying a univariate linear model to the PSD integrals.

The statistical analysis showed that the LDV was able to distinguish different flow levels in the envisioned range, as could the pressure probe, with a comparable sensitivity (Fig 7). We found that there seems to be very little energy in the signals beyond 200Hz, as displayed in Fig 8, where the EMMs rapidly decline in magnitude with increasing frequency.

Furthermore, our data suggest that the 0-200Hz range covers the frequency band within which to seek the footprint of the stenosis. As the instabilities are generated in the diverging region distal to the stenosis, one might expect that the highest sensitivity of the technique would be distal to the stenosis. Table 2 shows that this is confirmed by the statistical analysis. While the effect of increasing flow on the area under the PSD curve is significant for frequencies up to 200Hz for P_ICA_ and up to 150Hz for LDV_ICA_, the difference between zero flow and flow in P_CCA_ was no longer significant at 100Hz and for the LDV with a beam directed at the skin over the bifurcation, was significant only up to 50Hz. Therefore, a 100Hz threshold seems to make it possible to discern unstable post-stenotic flow from the more laminar flow of the CCA. This encouraging finding suggests that a frequency analysis of the LDV signals would allow us to discriminate between patients and healthy subjects, as a healthy carotid bifurcation is not subjected to stenosis-induced flow instabilities. Furthermore, based on our data, a 2ms temporal resolution would suffice to infer the presence of stenosis-induced flow instabilities. The currently used LDV default resolution (10μs) would hence not be necessary for this application. These findings would reduce drastically the amount of data storage required for in-vivo measurements, making the recordings faster to analyze hence allowing real-time processing in daily clinical practice.

**Table 2:** Significance level of the interaction term in Eq 1. Statistical significance (bold text, p < 0.01), confirms a dependence of the area under the PSD curve within the specified frequency range on the level of flow. This indicates the frequency bands containing relevant information for stenosis detection. Data are given for pressure measurements up- (CCA) and downstream (ICA) of the stenosis, and for LDV measurements up- (bifurcation) and downstream (ICA). Data shown are for the compliant model; all measurements with pressure catheter present.

	downstream		upstream	
Freq^.^Q_ICA_ significance	P_ICA_	LDV_ICA_	P_CCA_	LDV_bifurcation_
0–50 Hz	**2.79E-10**	**1.59E-08**	**1.59E-17**	**1.65E-05**
50–100 Hz	**3.07E-09**	**2.56E-07**	**6.55E-03**	2.26E-01
100–150 Hz	**2.80E-07**	**2.12E-03**	2.20E-01	5.82E-01
150–200 Hz	**1.26E-03**	5.84E-01	1.75E-01	3.68E-01
200–250 Hz	1.84E-01	9.90E-01	4.58E-04	2.94E-01
250–300 Hz	4.42E-01	9.52E-01	1.85E-02	3.60E-01
300–350 Hz	3.80E-01	9.89E-01	9.76E-01	2.71E-01
350–400 Hz	2.41E-01	9.26E-01	2.82E-03	6.64E-01
400–450 Hz	6.89E-01	9.73E-01	1.34E-01	9.63E-01

Measurement location, evaluated at several sites distal to the stenosis, came out as a significant factor in the statistical model, as reflected in the EMM values shown in Fig 8 (panel B). For P_ICA_, there is a clear decrease in the magnitude of the EMM as one moves further downstream from the stenosis, and thus away from the zone where flow disturbances are generated. The effect of measuring location on the PSD of the LDV was largely similar, except for one location (4D). We speculate that this is due to the complex wave propagation mechanics through the arterial walls and the gel. Location 4D was also closer to the distal wall of the Perspex box so probably more affected by reflections due to its stiff walls. Another effect that may also have played a role is the presence of the catheters, which was found to be significant. We speculate that the presence of the catheters, despite their modest dimensions, promoted the induction of flow instabilities through their obstructive effect (the downstream catheter had a nominal diameter of 1.5 mm and the cross-sectional area ratio hence ranged from 7.6% (based on A_downstream_ to 31.7% (based on A_stenosis_ Furthermore, the motion of the catheters, further amplifying the vibrations, might have played a role as well. As it was the purpose of this study to assess the congruence between LDV and pressure signals, considered as the gold-standard, there was no other option but to insert pressure catheters in the arterial lumen. The impact of the location on the displacement signals when the flow was not disturbed by the presence of the intra-arterial pressure probes (*probes = 0*) was found to be significant.

We performed experiments in both a compliant and a stiff model, with the stiff model measurements mainly performed as a basis for comparison with an ongoing CFD study that is beyond the scope of this experimental report. As is clear from Fig 3, signals were very different in both model settings, which translates into significantly different EMMs for the stiff and the compliant model. In particular, the stiff model was much more susceptible to wave reflections, visible in much stronger oscillations in pressure and LDV data that particularly increased the energy content of the signals in the 0-50Hz band. As expected, the amplitude of measured displacements was much lower in the stiff model than in the compliant model. The direct relevance of the stiff model experiments may be limited within the specific context of the assessment of LDV as a tool to screen for vibrations on the skin surface. Nevertheless, measurements do show a similar dependency of the PSD integral on the imposed flow level to that of the measurements in the compliant model, and were therefore included in the analysis. Including all available data led to an imbalance in the amount of data obtained from the compliant and stiff models in the statistical analysis. We verified, however, that all conclusions and relations between the two models are maintained when only considering data generated under physiological flow conditions and excluding all other data (Table 1, top 2 rows).

The values of Reynolds number used in this study spanned a wide range, with extreme values clearly beyond the physiological range in the carotid arteries [24]. This was done intentionally, as it was our specific aim to provoke flow instabilities and to run the model over a wide range of Reynolds numbers, encompassing laminar and highly turbulent flow conditions. The resulting average Reynolds number of all recordings was found to be Re = 1848. Using laser Doppler anemometry to investigate a 75% axisymmetric stenosis, it has been previously shown that, for a similar value of Re, turbulent flow was detected [31]. Note also that flow instabilities in the normal carotid arteries have been shown to arise at Reynolds number well below 1500, the lower limit of the commonly assumed threshold for transitional flows [32]. Secondly, water was used as test fluid for the experiments. This suited the purpose of the proof-of-concept study and is not expected to have an impact on the threshold value of Reynolds number for the onset of flow instabilities in the experimental setting. However, flow instabilities will only occur for in-vivo trans-stenotic flow levels that are 3 to 4 times higher than in this study. The impact of fluid viscosity on the intensity of flow instabilities and their onset is currently being investigated by means of a computational analysis, which will be reported in due course.

In this well controlled hydraulic bench study, the luxury of having a no-flow reference allowed us to improve the signal to noise ratio of the data and normalize PSD spectra to the no-flow condition. Measuring no-flow conditions was considered important as the recordings were not performed in an isolated noise-free laboratory, therefore the flow related features could have been missed among the background noise. Furthermore, showing the normalized PSD spectra of both LDV and pressure signals gave us to the chance to visually compare them with respect to their frequency-based behavior only. The comparison between the normalized and un-normalized data (namely the raw FFT data), however, showed no major differences in the predicted EMM trends for both P_ICA_ and LDV_ICA_. Besides the absolute value, the outcome of the statistical analysis for the un-normalized data, for the various locations examined on the compliant model with manometers present, does not differ substantially from the one of the normalized data. The lack of a no-flow reference would hence not be likely to have a major impact on the outcome of the analysis in-vivo.

An important limitation of this study is the fact that only one stenosed carotid geometry was tested (albeit extensively, covering a wide range of flow conditions and in a compliant and stiff material). While testing additional models with other degrees of stenosis (as well as those with no stenosis) and using different tissue-mimicking materials is certainly valuable, experimental testing using a variety of patient-specific carotid models, with different degrees of stenosis, differing geometries and different flow regimes, is time consuming and cumbersome to perform. For this reason we are presently carrying out a detailed high resolution computational analysis of the problem which can simulate a wide variety of patient-specific stenoses and with which it will be possible to estimate the sensitivity and specificity of this approach to the detection of occlusive carotid artery disease. The results of this study will be reported in due course. For now, we speculate that the critical parameters will be the degree of stenosis and its 3D geometry [33,34], combined with the level of trans-stenotic flow [35].

Overall, we conclude from this proof-of-concept study that the LDV is, in principal, able to recognize the presence of a stenosis–given that it produces flow instabilities. Note, however, that this study does not allow us to draw any conclusion regarding the critical level of stenosis that would be detectable with the methodology. Besides additional computational analysis, in-vivo measurements are currently in progress to assess the sensitivity and specificity of the envisioned technique.

## Abbreviations

ICA: Internal Carotid Artery
ECA: External Carotid Artery
CCA: Common Carotid Artery
LDV: Laser Doppler Vibrometry
Re: Reynolds number
FFT: Fast Fourier Transform
PSD: Power Spectral Density
EMMs: Estimated Marginal Means
